# *In situ* Forming Hyperbranched PEG—Thiolated Hyaluronic Acid Hydrogels With Honey-Mimetic Antibacterial Properties

**DOI:** 10.3389/fbioe.2021.742135

**Published:** 2021-11-16

**Authors:** Jeddah Marie Vasquez, Ayesha Idrees, Irene Carmagnola, Aa Sigen, Sean McMahon, Lennart Marlinghaus, Gianluca Ciardelli, Udo Greiser, Hongyun Tai, Wenxin Wang, Jochen Salber, Valeria Chiono

**Affiliations:** ^1^ Department of Mechanical and Aerospace Engineering, Politecnico di Torino, Turin, Italy; ^2^ Blafar Ltd., Dublin, Ireland; ^3^ Wenxin Wang Research Group, Charles Institute of Dermatology, University College Dublin, Dublin, Ireland; ^4^ Department of Surgery, Universitätsklinikum Knappschaftskrankenhaus Bochum, Ruhr-University, Bochum, Germany; ^5^ Department of Medical Microbiology, Ruhr-University Bochum, Bochum, Germany

**Keywords:** antibacterial, hyaluronic acid, hyperbranched PEG, thiol-ene click chemistry, honey-mimetic hydrogel, dressing, wound care

## Abstract

The rapidly increasing resistance of bacteria to currently approved antibiotic drugs makes surgical interventions and the treatment of bacterial infections increasingly difficult. In recent years, complementary strategies to classical antibiotic therapy have, therefore, gained importance. One of these strategies is the use of medicinal honey in the treatment of bacterially colonized wounds. One of the several bactericidal effects of honey is based on the *in situ* generation of hydrogen peroxide through the activity of the enzyme glucose oxidase. The strategy underlying this work is to mimic this antibacterial redox effect of honey in an injectable, biocompatible, and rapidly forming hydrogel. The hydrogel was obtained by thiol–ene click reaction between hyperbranched polyethylene glycol diacrylate (HB PEGDA), synthesized using reversible addition-fragmentation chain transfer (RAFT) polymerization, and thiolated hyaluronic acid (HA-SH). After mixing 500 µL HB PEGDA (10%, w/w) and 500 µL HA-SH (1%, w/w) solutions, hydrogels formed in ∼60 s (HB PEGDA/HA-SH 10.0–1.0), as assessed by the tube inverting test. The HB PEGDA/HA-SH 10.0–1.0 hydrogel (200 µL) was resistant to *in vitro* dissolution in water for at least 64 days, absorbing up to 130 wt% of water. Varying glucose oxidase (GO) amounts (0–500 U/L) and constant glucose content (2.5 wt%) were loaded into HB PEGDA and HA-SH solutions, respectively, before hydrogel formation. Then, the release of H_2_O_2_ was evaluated through a colorimetric pertitanic acid assay. The GO content of 250 U/L was selected, allowing the formation of 10.8 ± 1.4 mmol H_2_O_2_/L hydrogel in 24 h, under static conditions. The cytocompatibility of HB PEGDA/HA-SH 10.0–1.0 hydrogels loaded with different GO activities (≤ 500 U/L) at a constant glucose amount (2.5 wt%) was investigated by *in vitro* assays at 24 h with L929 and HaCaT cell lines, according to DIN EN ISO 10993-5. The tests showed cytocompatibility for GO enzyme activity up to 250 U/L for both cell lines. The antibacterial activity of HB PEGDA/HA-SH 10.0–1.0 hydrogels loaded with increasing amounts of GO was demonstrated against various gram-positive bacteria (*S. aureus and S. epidermidis*), antibiotic-resistant gram-positive bacteria (MRSA and MRSE), gram-negative bacteria (*P. aeruginosa*, *E. coli*, *and A. baumanii*), and antibiotic-resistant gram-negative strains (*P. aeruginosa and E. coli*) using agar diffusion tests. For all gram-positive bacterial strains, increasing efficacy was measured with increasing GO activity. For the two *P. aeruginosa strains*, efficacy was shown only from an enzyme activity of 125 U/L and for *E. coli* and *A. baumanii*, efficacy was shown only from 250 U/L enzyme activity. HB PEGDA/HA-SH 10.0–1.0 hydrogels loaded with ≤250 U/L GO and 2.5 wt% glucose are promising formulations due to their fast-forming properties, cytocompatibility, and ability to produce antibacterial H_2_O_2_, warranting future investigations for bacterial infection treatment, such as wound care.

## Introduction

The huge problem of antibacterial resistance to antibiotics has increased the demand for drug-free antimicrobial strategies, and this has led to reevaluation of ancient antibacterial remedies, such as those derived from natural products, including honey (Mandal and Mandal, 2011).

Research has been conducted on manuka (*L. scoparium*) honey, which has been demonstrated to be effective against several human pathogens, including *Escherichia coli* (*E. coli*), *Enterobacter aerogenes*, *Salmonella typhimurium*, *Staphylococcus aureus* (*S. aureus*), β-haemolytic streptococci, *vancomycin-resistant enterococci* (*VRE*), and *Pseudomonas aeruginosa* (*P. aeruginosa*) (Mandal and Mandal, 2011; Shenoy et al., 2012).

Honey has been widely studied for its bactericidal effects (Shenoy et al., 2012; Albaridi, 2019). Such antibacterial properties are attributed to many factors, including phytochemical components, osmotic effect of sugar on bacterial cells, acidic pH that helps macrophages in destroying bacteria, and the enzymatic activity of GO to produce *in situ* hydrogen peroxide from glucose (Bogdanov 1997; Abuharfeil et al., 1999; Cooper et al., 1999; Cooke et al., 2015). Particularly, hydrogen peroxide represents the main antimicrobial agent in honey: its concentration is determined by relative levels of glucose oxidase and glucose according to the following reaction (Wong et al., 2008; Mandal and Mandal, 2011):

$$\beta -D-Glucose\;+\;O_{2}\;\overset{Glucose\;Oxidase}{\to }\;D-gluconolactone+\;H_{2}O_{2}.$$

Reactive oxygen species (ROS), such as hydrogen peroxide, play a pivotal role in the orchestration of the normal wound-healing response. They act as secondary messengers to many immunocytes and nonlymphoid cells, which are involved in the repair process (Dunnill et al., 2017). Harnessing the potential of ROS to be used within wound care devices, provides the ability to accelerate the healing process and to prevent bacterial infection (Loo et al., 2012). There are several ROS-based technologies currently available in the market or applied in research for wound applications. Among them is SurgihoneyRO™, a commercially available antibacterial product based on a bioengineered honey gel, which enzymatically produces H_2_O_2_ from glucose oxidase and glucose after application to a wound (Cooke et al., 2015). Another ROS-based commercially available wound dressing is Oxyzyme (Moffatt et al., 2014). It consists of one hydrogel patch embedded with glucose, that is called the “wound contact patch” and a smaller hydrogel patch, called the “secondary wound patch,” embedded with GO enzyme. Such hydrogels are stacked on top of each other, then a cover dressing is applied to protect the wound from exposure. The *in situ* formed hydrogen peroxide from Oxyzyme allows the oxidation of loaded iodide ions into iodine, exerting antimicrobial activity, and the formation of oxygen, favoring wound healing (Brimson and Nigam, 2013).

Previously, a quick forming H_2_O_2_-releasing hydrogel was reported based on gelatin-hydroxyphenyl propionic acid, which triggered cross-linking by horseradish peroxidase enzyme (HRP)/ H_2_O_2_ (Lee et al., 2017). The amount of H_2_O_2_ not involved in hydrogel formation served as the antibacterial component and was gradually released. The hydrogel showed antimicrobial activity against gram-positive bacteria including *Staphylococcus aureus*, *S. epidermidis*, and clinical isolate of *methicillin-resistant S. aureus* (MRSA). As a drawback, residual H_2_O_2_ was only passively released by the system up to exhaustion, while the hydrogel was not able to continuously produce antimicrobial H_2_O_2_. Recently, Zhang et al. prepared a photocrosslinked antifouling hydrogel from polyethylene glycol diacrylate containing GO, to produce H_2_O_2_ in contact with glucose (Zhang et al., 2020). However, this hydrogel did not perfectly replicate antimicrobial honey-mimetic mechanisms as glucose was not added into the hydrogel.

Based on the current state of the art, this work aimed to produce an injectable, fast-forming, antibacterial hydrogel for future use in the treatment of wounds colonized or infected with bacteria that overcomes the limitations of other honey-mimetic products, such as the ability to adapt to each wound shape and release different amounts of H_2_O_2_ per hydrogel volume, depending on the individual wound situation. An ideal hydrogel for wound healing applications should form *in situ* within minutes under mild conditions. “Click” type chemical cross-linking was here exploited as a mechanism for rapid *in situ* formation of hydrogels (Kennedy et al., 2014). In detail, the hydrogel was formed by thiol–ene reaction between hyperbranched polyethylene glycol (PEG) diacrylate (HB PEGDA) and thiolated hyaluronic acid (HA-SH). HA was selected as it is one of the natural components of the dermal extracellular matrix (ECM) and has important functions during wound healing: it regulates inflammatory response and promotes fibroblast migration and proliferation (Dong et al., 2012; Loebel et al., 2017; Poldervaart et al., 2017). On the other hand, PEG is advantageous due to its nonimmunogenicity and resistance to protein adsorption (Dong et al., 2015). Thiol–ene crosslinking of the mixed system may impart to wound closure retard superior resistance to hyaluronidase-catalyzed degradation of HA and represents a tool to modulate hydrogel mechanical properties. Recently, Wenxin’s lab has prepared HB PEGDA/HA-SH hydrogels and exploited them to support adipose stem cells-based therapy, demonstrating their biocompatibility (Zhang et al., 2018).

In this work, HB PEGDA/HA-SH hydrogels were prepared from HB PEGDA solution (containing GO) and HA-SH solution (containing glucose, G). Physical encapsulation of GO may protect the enzyme from degradation at the wound site with respect to topical application (Arul et al., 2012). The interaction between GO and G causes the formation of antibacterial H_2_O_2_. By manipulating the concentrations of GO at constant G content within the hydrogel, the rate of formation and release of H_2_O_2_ was controlled. Loo et al. previously demonstrated that daily topical application of H_2_O_2_ solution (15 µL) with varying concentrations (10–166 mM) on skin wounds in C57/BL6 mice for overall 10 days caused different effects, from enhanced angiogenesis (10 mM concentration, i.e., 0.15 µmol/wound) to wound closure retard (166 mM concentration, i.e., 2.5 µmol/wound) (Loo et al., 2012). In this work, tuning the amount of GO in HB-PEGDA/HA-SH hydrogels at fixed G content allowed the release of suitable H_2_O_2_ concentrations per hydrogel volume within 24 h, which were able to control various gram-positive and gram-negative bacterial strains while largely avoiding cytotoxic effects in 2D cultures of L929 mouse fibroblasts and HaCaT human keratinocytes.

## Materials and Methods

### Materials

2,2′-Azobis(isobutyronitrile) (AIBN), tetraethylthiuram disulfide (TETD), polyethylene glycol diacrylate (PEGDA; Mw: 575 Da), butanone, hexane, diethyl ether, dimethyl formamide (DMF), acetone, lithium bromide (LiBr), glucose, glucose oxidase enzyme (E.C. 1.1.3.4), phosphate-buffered saline at pH 7.4 (PBS), Hank’s buffer solution, high-glucose Dulbecco’s modified Eagle’s medium (DMEM), foetal bovine serum (FBS), resazurin sodium salt, and penicillin-streptomycin (PS) were supplied from Sigma-Aldrich, United Kingdom. Thiolated hyaluronic acid (HA-SH, 80% degree of thiolation, 400 kDa) was provided by Blafar, Ireland.

### Synthesis of Hyperbranched PEG-Diacrylate

**FIGURE 1:**
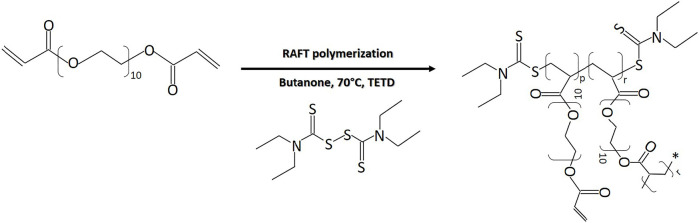
RAFT polymerization reaction of PEG-diacrylate for HB PEGDA synthesis (*p* = 0.45) (*r* = 0.55).

HB PEGDA was synthesized *via* reversible addition-fragmentation chain transfer (RAFT) polymerization, through the reaction illustrated in Figure 1. Briefly, in a 250 ml double-neck flask, the following reagents were dissolved in 160 ml butanone: 46 g PEGDA, 948.92 mg TETD (chain transfer agent), and 735.66 mg AIBN (reaction initiator). The double-neck flask was sealed and the reaction solution was purged with argon; afterward, the reaction vessel was placed into an oil bath at 70°C for 2–3 h.

Samples were taken hourly to be analyzed by gel permeation chromatography/size exclusion chromatography (Agilent 1260 Infinity GPC/SEC system, Agilent Technologies, Santa Clara, CA, United States) to monitor the molecular weight increase of the polymer and the molar conversion. When the molecular weight reached 10–20 kDa, polymerization was stopped by quenching the flask with ice water and exposed to the air. The polymer was purified by precipitation in diethyl ether: hexane solution (2:1 v/v) three times and then dried in a vacuum oven for 48 h to remove residual solvent.

### Chemical Characterization of HB PEGDA

#### GPC/SEC Analysis

Samples were prepared for GPC analysis (Agilent PL-GPC50) by dissolving 3 mg of HB PEGDA in 1 ml of DMF. Each solution was filtered using a syringe filter type Sartorius Minisart™ (glass fibre upstream filter combined with a 0.45 µm pore size cellulose acetate filter, Fisher Scientific, United Kingdom) into a small amber GPC vial. The samples were analyzed by GPC/SEC to monitor number average molecular weight (Mn), weight average molecular weight (Mw), polydispersity index (PDI = Mw/Mn), and the degree of polymerization (PD) as the reaction proceeded. The system was equipped with a refractive index detector (RI). The columns in series (30 cm PLgel Mixed-C, two in series) were eluted using DMF (containing 0.1% LiBr) as the mobile phase and calibrated with poly (methyl methacrylate) (PMMA) standards. All calibrations and analysis were performed at 60°C and with a flow rate of 1 ml min^−1^.

#### ^1^H Nuclear Magnetic Resonance

For characterizing molecular structure, ^1^H nuclear magnetic resonance (NMR) was used. The polymer samples were dissolved in chloroform-D (CDCl_3_) and ^1^H NMR analysis was carried out on a 400 MHz Varian NMR spectroscopy system. Data were analyzed using MesReNova 6.1 processing software. The chemical shifts were referenced to the lock chloroform (CDCl_3_, 7.26 ppm).

The branching ratio, i.e. the ratio of the branched to all PEGDA units, was calculated by the following equation (Dong et al., 2015):

$$Branching\;ratio=1-\frac{Linear\;PEGDA\;units}{All\;PEGDA\;units}=1-\;\frac{Integral\;of\;y}{Integral\;of\;d/4},$$

with y indicating the chemical shifts of pendant acrylate groups (in the 5.8–6.4 ppm range) and d indicating the chemical shifts of PEG groups (3.95–4.34 ppm range).

Furthermore, vinyl ratio, i.e., the amount of vinyl groups (mol%) was also calculated (Zhang et al., 2018; Wang et al., 2015):

$$Vinyl\;ratio=\frac{Linear\;PEGDA\;units}{All\;PEGDA\;units}=\;\frac{Integral\;of\;y}{Integral\;of\;d/4}.$$

### Preparation of the HB PEGDA/HA-SH 10.0–1.0 Hydrogel

HB PEGDA/HA-SH 10.0–1.0 hydrogel formulations for the different physicochemical and biological characterizations were always freshly prepared for each experiment by homogeneously mixing equal volumes of HB PEGDA/1X PBS solution (40% w/w) and HA-SH/1X PBS solution (4% w/w) by vortexing vigorously.

### Gelation Time Measurement

The gelation time for HB PEGDA/HA-SH 10.0–1.0 hydrogels was measured by the vial tilt method (Deshmukh et al., 2010). Briefly, 500 µL HB PEGDA solution (40% w/w) was combined with 500 µL HA-SH solution (4% w/w). The vials were tilted upside down and observed for 5 s. Gelation was considered complete when no flow was observed within 5 s. Measurements of gelation time were carried out in triplicate (*n* = 3).

### Water Uptake of Hydrogels

In order to determine the water uptake profile, 200 µL HB PEGDA/HA-SH 10.0–1.0 hydrogel was placed in a preweighed 20 ml scintillation vial and initially weighed. Then, 2 ml 1X PBS was added to the vial and incubated at 37°C. At different time points up to 6 weeks (every 2 h during the first 8 h, every 24 h for the first week, and every 2 days onward), PBS was removed and the vials were weighed again. After weight measurement, 2 ml fresh PBS solution was added again and the vial was replaced in the incubator. Measurements were performed in quadruplicates (*n* = 4).

Water uptake percentage was calculated as follows:

$$Water\;uptake\;\left(\%\right)=\;\frac{W_{t}-W_{0}}{W_{0}}\times 100,$$

where *W*_*t*_ is the weight of the hydrogel at time point t and *W*_*0*_ is the initial weight of the hydrogel.

### Preparation of Honey-Mimetic HB PEGDA/HA-SH Hydrogels by GO and G Encapsulation

The H_2_O_2_-releasing hydrogel samples were prepared by mixing HB-PEGDA solution (40% w/w), containing various GO concentrations, with HA-SH (4% w/w) solution, containing constant G concentration (Supplementary Figure S1).

In detail, HB-PEGDA was dissolved in 1X PBS to prepare a 80% (w/w) stock solution. 1 U is the amount of GO that catalyzes the conversion of 1 μM glucose into 1 μM H_2_O_2_ within 1 min [Nomenclature Committee of the International Union of Biochemistry (NC-IUB), 1979]. The purchased GO was a lyophilized product with 19.290 U/mg activity. Enzyme stock solution was prepared as 10,000 U/L in 1X PBS. Enzyme solutions (Solution A, shown in Table 1; Supplementary Figure S1) were prepared from the enzyme stock solution by dilution with PBS. Same volumes of Solution A and HB-PEGDA solution (80% w/w) were mixed to obtain Solution B (Table 1; Supplementary Figure S1). HA-SH solution (4% w/w, Solution D, Supplementary Figure S1) containing 5% (w/w) G (Solution C, Supplementary Figure S1) was prepared. HB PEGDA/HA-SH 10.0–1.0 hydrogels with various GO concentrations and 2.5% w/w content were prepared (Table 1; Supplementary Figure S1).

**TABLE 1:** HB PEGDA/HA-SH 10.0–1.0 hydrogels containing GO and G.

GO solution in PBS (U/L) (Solution A)	GO concentration in HB PEGDA solution (40% w/w) (U/L) (Solution B)	G concentration in HA-SH solution (4% w/w) (w/w%) (Solution C)	GO concentration in the HB PEGDA/ HA-SH 10.0–1.0 hydrogel (U/L)
0	0	5	0
100	50	5	25
200	100	5	50
500	250	5	125
1,000	500	5	250
2,000	1,000	5	500

For further investigations, hydrogel test specimens were prepared by mixing equal volumes (e.g., 15 µL) of Solution B with Solution C. Afterward, properly dispersed mixture was completely pipetted onto a Teflon™ plate surface. Following this protocol, semispherical hydrogel beads with ∼5 mm diameter were prepared. The HB PEGDA/HA-SH 10.0–1.0 hydrogel samples were named according to the concentration of encapsulated GO: PEGDA/HA-SH 10.0–1.0 500, 250, 125, 50, and 25 U/L GO, respectively.

### Analysis of Hydrogen Peroxide Released From HB PEGDA/HA-SH 10.0–1.0 Hydrogels

Hydrogels with different compositions (Table 1) were formed by mixing a volume of 100 µL HB PEGDA/GO solution (Solution B) and 100 µL HA-SH/G solution (Solution C) into a 1 ml Eppendorf tube. After the hydrogel was formed, 800 µL of deionized H_2_O was added to the 200 µL hydrogel sample and incubated at 37°C. The formation of H_2_O_2_ was monitored at different time points between 2 and 24 h. The production of H_2_O_2_ was quantified by a colorimetric pertitanic acid assay (Sigma-Aldrich, United Kingdom) according to the method by Eisenberg (1943). Briefly, 100 µL of the release solution (containing *in situ* generated H_2_O_2_) was withdrawn at specific time points and placed in a 96-well plate. Then, 50 µL of the assay solution (2.5% w/w titanium oxysulfate in 2 M H_2_SO_4_ solution) was added. The color of the solution changed into yellow by H_2_O_2_ reaction with Ti(IV) (Supplementary Figure S2), according to the reaction:

$$Ti^{4+}+H_{2}O_{2}+2H_{2}O\to H_{2}TiO_{4}\left(\mathrm{pertitanic}\;\mathrm{acid},yellow\right)+4H^{+}.$$

The amount of pertitanic acid formed, corresponding to the amount of H_2_O_2_ released by hydrogels, was derived from the absorbance intensity of the pertitanic acid solution at 407 nm wavelength, measured in a SpectraMax M3 multi-mode microplate reader (Molecular Devices, San José, CA, United States) equipped with SoftMax Pro software for data collection and analysis. For obtaining the calibration curve, 100 µL of H_2_O_2_ solutions with 0–20 mM concentrations were pipetted into the wells of a 96-well plate. Then, 50 µL of the prepared ROS assay solution was added into each well. The plate was shaken in the plate reader for 15 s before the absorbance intensity was read at 407 nm wavelength. Based on the calibration curve (Supplementary Figure S3), H_2_O_2_ concentration in the release medium was calculated as follows:

$$H_{2}O_{2\;}\left[mM\right]=\;\frac{Absorbance\;-\;0.0724}{0.1777}.$$

Considering that this is the H_2_O_2_ concentration released by 200 µL hydrogel in 800 µL release medium, H_2_O_2_ amount (mmol) per volume hydrogel was calculated through the following equation:

$$\frac{H_{2}O_{2\;}\left(mmol\right)}{Volume_{hydrogel}\left(L\right)\;}=\;\frac{Absorbance\;-\;0.0724}{0.1777}\times 4.$$

Each measurement was performed in quadruplicate (*n* = 4). H_2_O_2_ concentration values were reported in mM, i.e., mmol H_2_O_2_ released per unit volume (L) of hydrogel.

### Cytocompatibility Assessment of Hydrogels

Cell lines including L929 (a murine fibroblast cell line) and HaCaT (a transformed human keratinocyte cell line) were obtained from DSMZ (German Collection of Microorganisms and Cell Cultures). L929 cells were maintained in RPMI 1640 cell culture medium with stable glutamine without glucose (P04-18500, PAN Biotech, Germany), containing 10% foetal bovine serum (FBS; PAN Biotech, Germany) under physiological culture conditions (37°C, 5% CO_2_), and subcultured using 0.25% trypsin (Gibco, ThermoFisher Scientific, Germany). HaCaT cells were maintained in Dulbecco’s modified Eagle’s medium (DMEM) cell culture medium (PAN Biotech, Germany) containing 10% FBS under physiological culture conditions (37°C, 5% CO_2_), and subcultured using TrypLE™ Express (ThermoFisher Scientific, Germany).

Defined aliquots of cell suspensions for each cell type were used as follows: L929 [passage number (P) 7–P10] 0.7 × 10^5^ cells/well and HaCaT (P35–P37) 2.5 × 10^5^ cells/well were pipetted into 24-well plates. Cells were incubated at 37°C, with 5% CO_2_ for 24 h. Cell culture subconfluency and cell morphology were verified before exposing the cells to hydrogel samples. The culture medium was removed and replaced with fresh medium (1 ml) before starting the assessment.

HB PEGDA/HA-SH 10.0–1.0 hydrogel samples (30 µL) containing G and GO (Table 1) were freshly prepared under sterile conditions right before exposing them to the cell monolayer culture either through direct or indirect contact method (according DIN EN ISO 10993-5). For direct contact test, freshly prepared hydrogel samples (Table 1) were equilibrated in respective cell culture media for 10 min and placed in the center of the cell monolayer culture without making unnecessary movements of the specimens. Thus, each hydrogel sample of ∼5 mm diameter covered approx. 1/3rd growth area of the well surface of a 24-well cell culture plate.

For indirect contact test, the hydrogel samples were exposed to L929 and HaCaT cells through 24-well Transwell® inserts (PET membrane, 1 μm pore size, 6.5 mm diameter, Corning®, Sarstedt, Germany), in a 24-well cell culture plate. Cell culture media volumes at apical (250 μL) and basal sides (800 μL) of inserts were maintained at the same level outside and inside the Transwell inserts. Cells on tissue culture treated polystyrene (TCPS) wells, without the addition of hydrogel samples, were used as “TCPS control wells,” while cells treated with lysis solution (9% Triton® X-100 in water from Promega, Germany) served as “lysis control.” Cells treated with HB PEGDA/HA-SH 10.0–1.0 hydrogels neither containing GO (0 U/L GO) nor glucose were also used as “polymer control”. The well plate was then incubated at 37°C, with 5% CO_2_ for 24 h.

After 24 h, the supernatant culture medium and HB PEGDA/HA-SH 10.0–1.0 specimens were carefully removed, and the CellTiter-Blue® assay (CTB, Promega, Germany) was performed for measuring the cell viability according to Promega’s standard protocols (O’Brien et al., 2000).

To this aim, 400 μL CTB reagent (enough to cover the surface of each well in a 24-well plate) was pipetted into each well and left in incubation for 2 h at 37°C and 5% CO_2_. The cell supernatant was transferred to black microtiter 96-well plates. Fluorescence intensity was measured at excitation (Ex) of 560 nm and emission (Em) of 590 nm using a TECAN microplate reader Infinite® 200 PRO (Tecan, Switzerland).

### Morphological Analysis of Cells

Changes in morphological appearance and visualization of live and dead L929 and HaCaT cells were evaluated after a culture time of 24 h using bright field and fluorescent microscopy (Olympus IX51, Germany). Fluorescent staining was performed through Live/Dead imaging (Live/Dead Cell Staining Kit II, Promokine, VWR, Germany) using calcein-AM and ethidium homodimer III (EthD-III) to detect live (green-fluorescence for live cells by enzymatic conversion of nonfluorescent substrate calcein-AM, Ex/Em ∼495 nm/∼515 nm) and dead (red-fluorescence for dead cells upon binding to nucleic acid, Ex/Em ∼530 nm/∼635 nm) cells.

### Antibacterial Activity

*Staphylococcus aureus* (ATCC 29213), *Staphylococcus epidermidis* (ATCC 12228), *Pseudomonas aeruginosa* (ATCC 27853), *Escherichia coli* (ATCC 25922), and *Acinetobacter baumannii* (as some of the most relevant bacteria in infected wounds) were used to evaluate antibacterial activity of H_2_O_2_-releasing HB PEGDA/HA-SH 10.0/1.0 hydrogels containing different amounts of GO (25–500 U/L, Table 1; Supplementary Figure S1) and constant 2.5% w/w G. Additionally, resistant strains with most commonly found resistance phenotypes were also tested including *methicillin-resistant Staphylococcus aureus* (MRSA), *methicillin-resistant Staphylococcus epidermidis* (MRSE), VIM-2–producing drug-resistant *Pseudomonas aeruginosa* (Verona integron-encoded metallo- (VIM-2) β-lactamases-producing multidrug-resistant *P. aeruginosa*) (Viedma et al., 2012), and KPC-2-producing drug-resistant *Escherichia coli* (Ben Tanfous et al., 2017). These clinically isolated and highly resistant gram-negative bacteria came from the state reference sample and database for gram-negative bacteria at the Department for Medical Microbiology, Ruhr-University Bochum. The disc-diffusion test was performed for antimicrobial susceptibility testing according to EUCAST (European Committee on Antimicrobial Susceptibility Testing) guidelines (EUCAST 2016). Bacterial strains were revived and cultured on fresh Mueller-Hinton (MH) agar 1 day before testing. The well-isolated colonies were suspended in 0.9% normal saline and adjusted to McFarland 0.5 (Weinstein 2021). The inoculum was spread evenly over the entire surface of MH agar using a sterile cotton swab. The freshly prepared hydrogel samples (30 μL), in the form of ∼5 mm diameter bead-shaped discs, were applied on the agar plate within 15 min of bacterial inoculation. The plates were then incubated at 37°C for 16–20 h (within 15 min of disc application). The zone of inhibition (ZOI, in mm) was measured using a scale bar by reading the MH plates from the back against a dark background and zone edges were read at the point of complete inhibition.

### Statistical Analysis

Data are represented as mean ± standard deviation (SD) from two to four replicates depending on the analysis. Comparisons were made using one-way ANOVA or independent variable Student’s *t*-test. Observations were considered to be significantly different for *p* < 0.05 and highly significant for *p* < 0.01.

All cell culture experiments and analyses of antibacterial activity were performed independently in duplicate (*N* = 2, number of independent experiments) and four technical replicates were made and examined per experiment (*n* = 4).

## Results

### Chemical Characterization of HB PEGDA

The HB PEGDA polymer was synthesized with weight average molecular weight (Mw) of 16,656 Da, a number average molecular weight (Mn) of 11,060 Da, a polydispersity index (PDI) of 1.5, and a molar conversion of 32.5%, as assessed by GPC analysis.

^1^H NMR spectra (Supplementary Figure S4) allowed the analysis of the branched structure and amount of pendant acrylate groups in HB PEGDA. The pendant acrylate groups were identified through the three characteristic chemical shifts at 6.4–5.8 ppm, while PEG groups were identified through the chemical shifts at 4.34–3.95 ppm. Vinyl ratio was measured to be 57 mol% with a vinyl content of 0.99 mmol vinyl groups/g polymer. On the other hand, branching ratio was 43 mol%.

### HB PEGDA/HA-SH 10.0–1.0 Hydrogels: Formation Mechanism and Gelation Time

**FIGURE 2:**
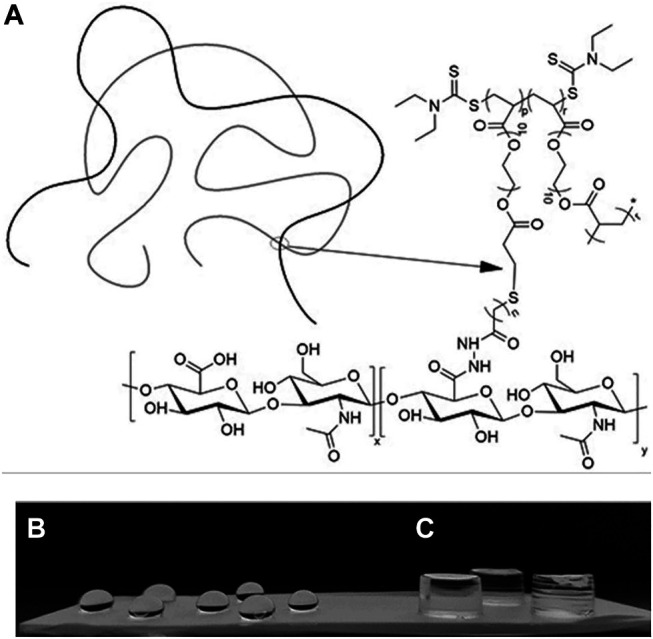
**(A)**: Schematic representation of the formation of a HB PEGDA/HA-SH hydrogel by thiol–ene cross-linking mechanism. **(B)** *:* HB PEGDA/HA-SH 10.0–1.0 hydrogel beads (30 μL) on the Teflon™ plate surface. **(C)** *:* HB PEGDA/HA-SH 10.0–1.0 hydrogels (200 μL) with cylindrical shape obtained by hydrogel cross-linking in a convenient mold. (*p* = 0.45) (*r* = 0.55) (*n* = 2) (*x* = 0.58) and (*y* = 0.42).

The HB PEGDA/HA-SH 10.0–1.0 hydrogel formed through the reaction of acrylate groups of HB PEGDA with thiol groups of HA-SH via Michael addition reaction. Figure 2A schematically illustrates HB PEGDA and HA-SH cross-linking while Figures 2B,C show photos of HB PEGDA/HA-SH 10.0–1.0 hydrogels with different shapes. A volume of 30 µL HB PEGDA/HA-SH 10.0–1.0 solution poured onto a hydrophobic Teflon™ plate surface formed hydrogel beads with ∼5 mm size at their bottom side (Figure 2B). HB PEGDA/HA-SH 10.0–1.0 hydrogels with 200 µL volume and cylindrical shape were also formed using convenient molds (Figure 2C). The tube inverting test showed that HB PEGDA/HA-SH 10.0–1.0 hydrogels (with total volume of 1 ml) have an average gelation time of 63 s.

### Water Uptake of HB PEGDA/HA-SH 10.0–1.0 Hydrogels

**FIGURE 3:**
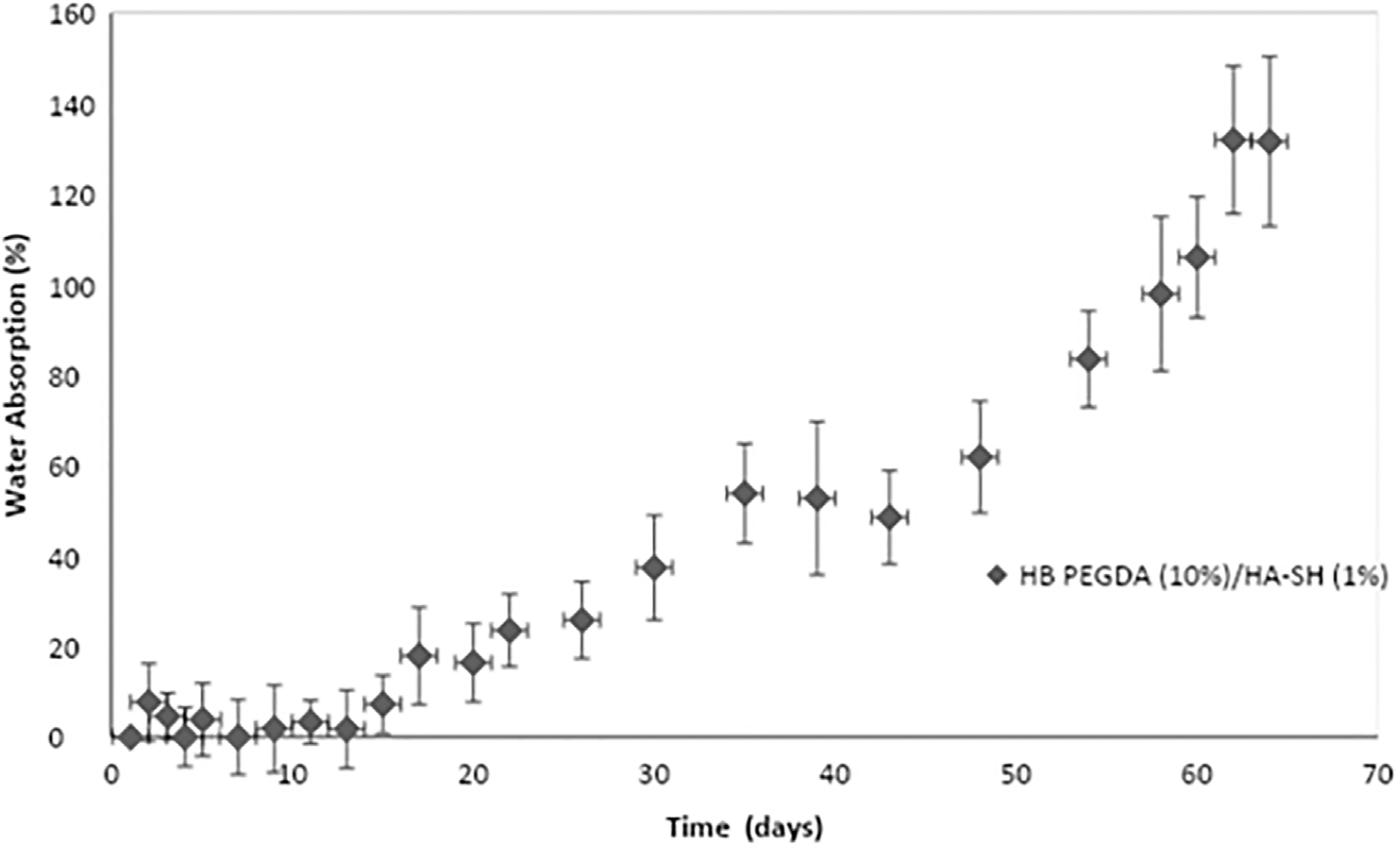
Water uptake percentage of HB PEGDA/HASH 10.0–1.0 hydrogels as a function of time. Tests were performed using the HB PEGDA/HASH 10.0–1.0 hydrogel (200 μL) in 2 ml PBS solution at 37°C (*n* = 4).

Figure 3 shows water uptake percentage as a function of time for HB PEGDA/HA-SH 10.0–1.0 hydrogels (200 µL). In the first 2 weeks, absorbed water was less than 10% of hydrogel initial mass. After 14 days, water uptake percentage tended to increase as a function of time. However, HB PEGDA/HA-SH 10.0–1.0 hydrogel did not dissolve up to 64 days, reaching a water uptake percentage of ∼130%.

### Hydrogen Peroxide Release From HB PEGDA/HA-SH 10.0–1.0 Hydrogels

**FIGURE 4:**
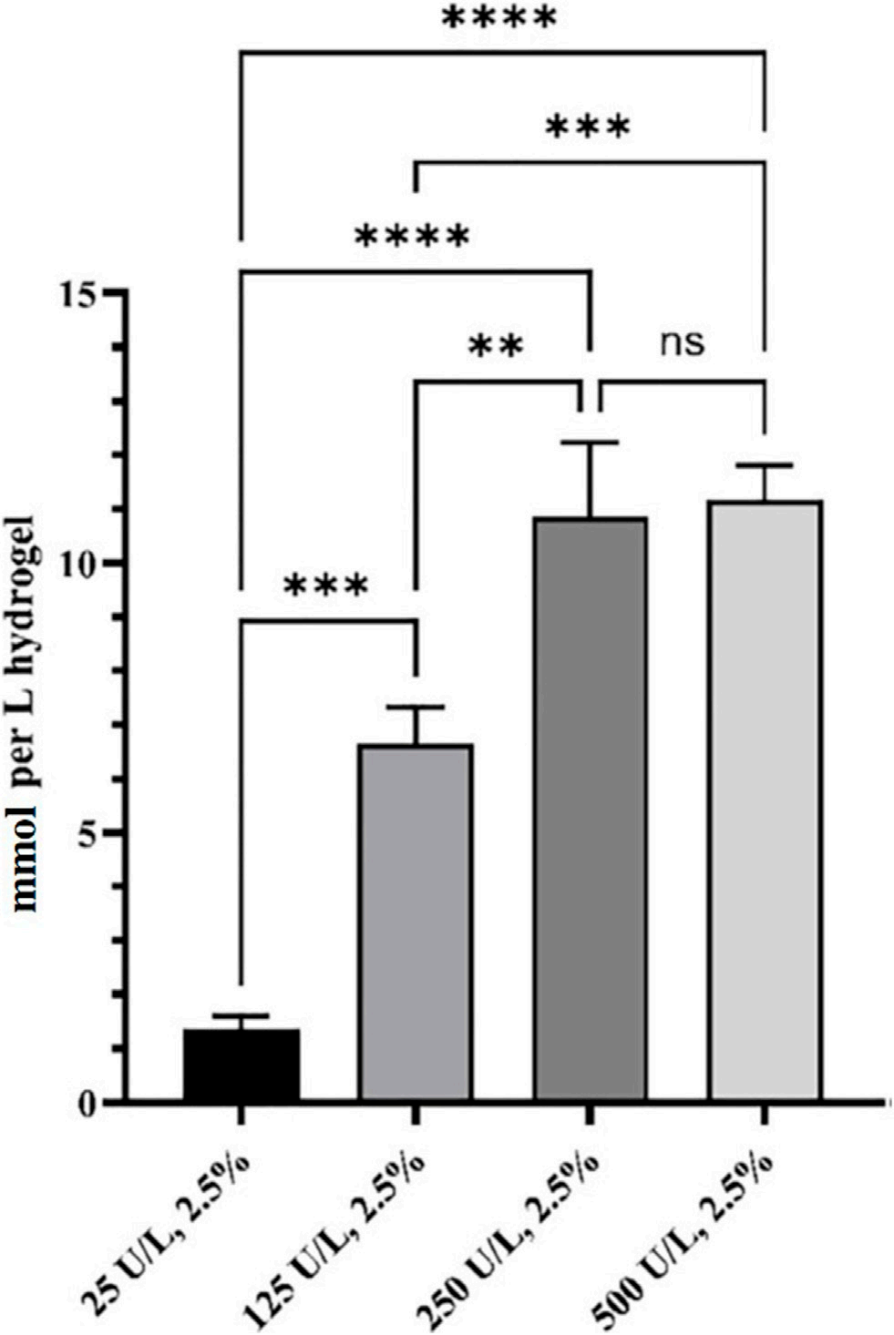
Released H_2_O_2_ from HB PEGDA/HA-SH 10.0–1.0 hydrogels containing 2.5% w/w G and different amounts of GO (0–500 U/L), expressed as mmol/L of hydrogel. Release data were obtained under static conditions over 24 h (*n* = 4). (** indicates *p* value = 0.013; *** indicates *p* value = 0.0008; **** indicates *p* value < 0.0001).

The *in situ* production and release of hydrogen peroxide from HB PEGDA/HA-SH 10.0–1.0 hydrogels (with 200 µL volume) with varying GO concentrations from 25 to 500 U/L and constant G amount (2.5% w/w) (Table 1) was quantified over 24 h (Figure 4).

The amount of *in situ* released hydrogen peroxide increased continuously and significantly from 1.4 ± 0.2 to 10.8 ± 1.4 mmol per L hydrogel as a function of GO concentration (from 25 to 250 U/L). By further increasing the amount of GO to reach 500 U/L activity, no further increase in the amount of *in situ* released hydrogen peroxide was measured. In fact, the H_2_O_2_ released from the hydrogel HB PEGDA/HA-SH 10.0–1.0 with 500 U/L GO and 2.5% w/w G was 11.2 ± 0.7 mmol per L hydrogel, which was a nonsignificantly different amount with respect to what released from the hydrogel with 250 U/L GO and 2.5% w/w G (*p* < 0.001).

### Cytotoxicity Analysis

#### Direct and Indirect Contact Test With L929 Fibroblasts

**FIGURE 5:**
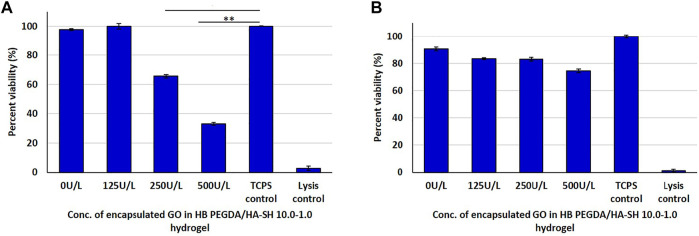
Post 24 h cell viability data of L929 cells interacting with H_2_O_2_-releasing HB PEGDA/HA-SH 10.0–1.0 hydrogels containing different amounts of GO (25–500 U/L) and constant G amount (2.5% w/w) using the quantitative CTB assay: **(A)** direct contact: 250 U/L and TCPS control (* indicates *p* value = 0.0006); 500 U/L and TCPS control (** indicates *p* value = 0.0001); **(B)** indirect contact tests.

In the direct contact method with L929 cells, CTB assay results showed a decrease in cell viability with increasing GO amount, due to progressively higher H_2_O_2_ release (Figure 5A). The same type of hydrogel samples were also exposed to L929 cells via an indirect contact test. The viability of L929 cells (Figure 5B) was higher in indirect assays (∼75–83%)—with cells exposed to the H_2_O_2_ amount released from hydrogel samples—than in direct assays (∼37–100%)—with cells in contact with H_2_O_2_-releasing hydrogels (Figure 5A).

#### Direct and Indirect Contact Test With HaCaT Keratinocytes

**FIGURE 6:**
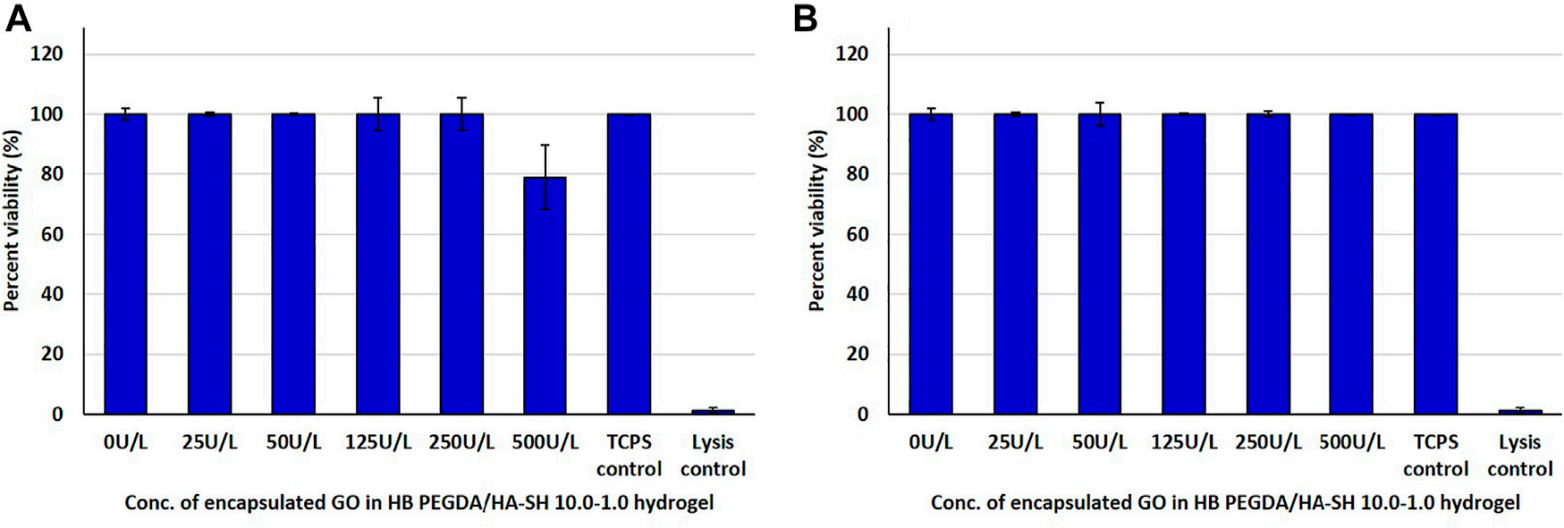
Post 24 h cell viability data of HaCaT cells interacting with H_2_O_2_-releasing HB PEGDA/HA-SH 10.0–1.0 hydrogels containing different amounts of GO (25–500 U/L) and constant G amount (2.5% w/w) using the quantitative CTB assay: **(A)** direct contact and **(B)** indirect contact tests.

HaCaT cells in direct contact with hydrogels containing GO concentrations from 25 to 250 U/L showed similar viability to control cells. However, the viability of HaCaT cells in contact with hydrogels with GO concentration of 500 U/L decreased to approximately 80% with respect to control cells (Figure 6A). In indirect tests, the viability of HaCaT cells exposed to eluates from HB PEGDA/HA-SH 10.0–1.0 hydrogels with different GO concentrations was similar to control cells (Figure 6B).

### Analysis of L929 and HaCaT Cell Morphology in Direct Contact Assays

#### Direct Contact Test With L929 Fibroblasts

On TCPS, after an incubation period of 24 h, the L929 fibroblasts showed a typical spread morphology with only a few rounded cell bodies that were either in division (in pairs) or apoptotic (isolated) (Supplementary Figure S5A). In the lysis control, significantly few cell bodies were seen compared to those on the TCPS surface, which were also more separated from each other and had fewer spread cell bodies (reduced nucleus–plasma relation) with mostly roundish morphology. Morphology was similar for cells in contact with control hydrogel samples (not containing GO and G), hydrogels containing 25–125 U/L GO at constant G content and control TCPS. In the case of HB PEGDA/HA-SH 10.0–1.0 sample with 250 U/L GO, most L929 fibroblasts showed similar morphology to TCPS controls; however, a few less densely packed cells with roundish morphology were also detected. Finally, in the case of HB PEGDA/HA-SH 10.0–1.0 sample with 500 U/L GO significantly fewer adherent cells were observed, and they almost exclusively exhibited a round morphology similar to those in the lysis control (Supplementary Figure S5A).

Live/dead assays served as evidence for almost 100% viability of L929 fibroblasts after 24 h cultivation on the control TCPS surface. Indeed, green-fluorescence results from the conversion of calcein-AM to calcein by esterase activity in viable cells. In contrast, only red-fluorescent avital cells were detected after treatment with Triton® X-100 (lysis control) (Supplementary Figure S5B). If the cell membrane integrity is disturbed or becomes porous and cells die, the intercalation dye EthD-III can penetrate the cells, enter their nucleus, and interact with their DNA. The Viability of L929 fibroblasts in contact with HB PEGDA/HA-SH 10.0–1.0 control hydrogel and TCPS controls was similar, showing rare red-fluorescent avital cells. However, L929 fibroblasts were slightly less close together than in control samples in some areas of the representative images. Similar results were obtained for cells in contact with HB PEGDA/HA-SH 10.0–1.0 hydrogel samples containing 25–50 U/L GO at constant G loading. On the contrary, only a few L929 fibroblasts in contact with hydrogel samples with 125 U/L GO activities showed prevalent green-fluorescent staining and normal size, while most cells showed reduced size and copresence of both staining. Finally, rare green-fluorescent cells were observed in contact with samples with 250 U/L GO activities, while most areas showed cells with double staining. When the amount of GO was further increased to 500 U/L, the removal of the hydrogel sample, the rinsing step and the staining showed a significantly reduced number of cells, with approximately equal number of vital and avital cells (Supplementary Figure S5B).

#### Direct Contact Test With HaCaT Keratinocytes

HaCaT keratinocytes showed typical spread morphology on TCPS controls after an incubation period of 24 h (Supplementary Figure S6A). A confluent monolayer of human HaCaT keratinocytes was observed, with relatively uniform nucleus–plasma ratio, forming a relatively uniform, hexagonal pattern in their entirety. Each cell was in very close contact with six neighboring cells. In the case of HaCaT cells treated with Triton® X-100, fewer cells remained on TCPS plates, separated from each other, showing clearly shrunken cell bodies. In contrast, the morphology of HaCaT keratinocytes in contact with control hydrogels and hydrogels with 25–250 U/L GO and constant G amount was similar to that of cells on control TCPS plates. On the other hand, only fewer adherent cells with the typical HaCaT morphology were observed after the contact with hydrogels containing 500 U/L GO (Supplementary Figure S6A).

Live/dead fluorescence staining confirmed viability of HaCaT cells in contact with hydrogels containing up to 250 U/L GO. In contrast, many attached, but avital cells were observed in the case of HaCaT cells in contact with hydrogels containing up to 500 U/L GO (Supplementary Figure S6B).

### Antibacterial Testing

**FIGURE 7:**
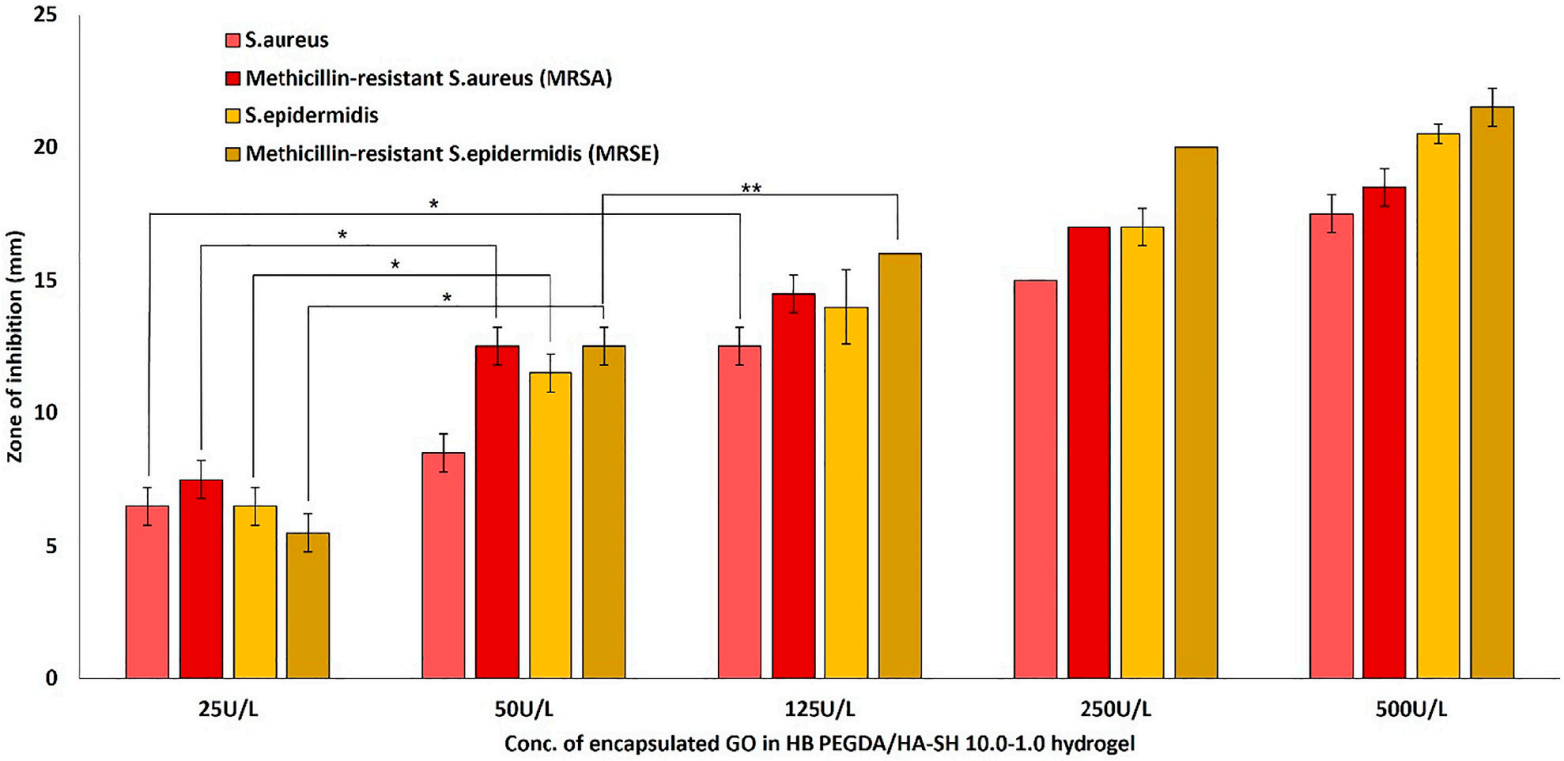
Antibacterial activity of *in situ* H_2_O_2_-releasing HB PEGDA/HA-SH 10–1.0 hydrogels containing different GO activities (25–500 U/L) at constant G amount of 2.5 wt% against Gram-positive bacteria *S. aureus* and *S. epidermis* and two antibiotic-resistant clinical isolates of MRSA and MRSE. The graph shows the measured inhibition zones (ZOI) of the agar diffusion assays. (*p < 0.01; **p < 0.05).

**FIGURE 8:**
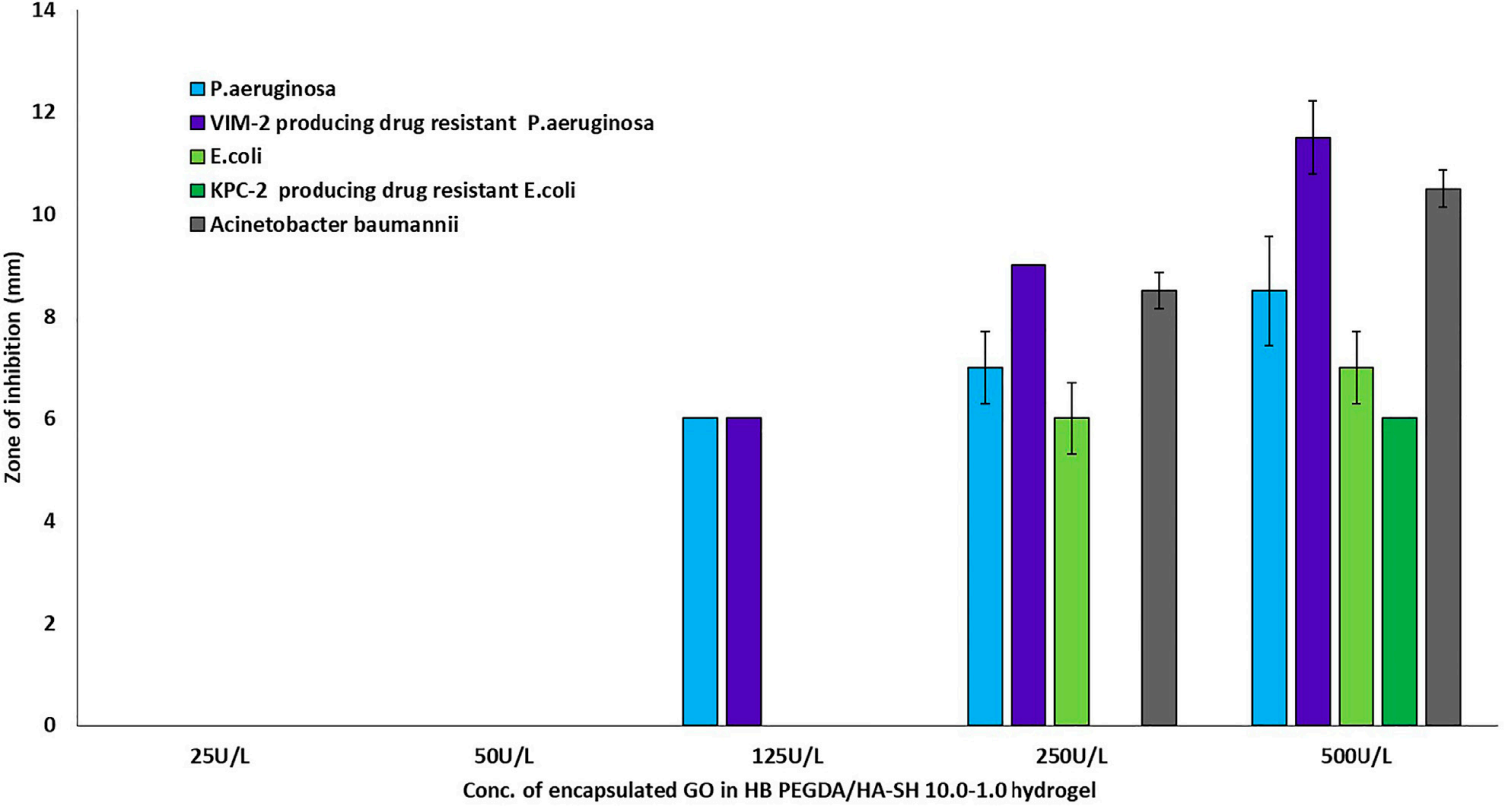
Antibacterial activity of *in situ* H_2_O_2_-releasing HB PEGDA/HA-SH 10–1.0 hydrogels containing different GO activities (25–500 U/L) at constant G amount of 2.5 wt% against gram-negative bacteria *P. aeruginosa*, *E. coli*, and *A. baumanii*, and two highly antibiotic-resistant clinical isolates VIM-2-producing drug -resistant *Pseudomonas aeruginosa* and KPC-2-producing drug-resistant *Escherichia coli*. The graph shows the measured inhibition zones (ZOI) of the agar diffusion assays.

The antimicrobial activity or efficacy of the novel honey-mimetic H_2_O_2_-releasing HB PEGDA/HA-SH 10.0–1.0 hydrogels containing different amounts of GO (25–500 U/L) and constant G amount (2.5% w/w) was investigated against a variety of gram-positive (Figure 7) and gram-negative (Figure 8) bacteria that have significant clinical importance in wound colonization and infection.

In addition to *Staphylococcus aureus* (ATCC 29213), *Staphylococcus epidermidis* (ATCC 12228), *Pseudomonas aeruginosa* (ATCC 27853), *Escherichia coli* (ATCC 25922), *Acinetobacter baumannii* (some of the most relevant bacteria in infected wounds), and some of the most commonly found resistance phenotypes, including *methicillin-resistant Staphylococcus aureus* (MRSA), *methicillin-resistant Staphylococcus epidermidis* (MRSE), VIM-2-producing drug-resistant *Pseudomonas aeruginosa*, and KPC-2-producing drug-resistant *Escherichia coli*, were also tested.

All gram-positive bacteria types showed susceptibility (appearance of an inhibition zone, ZOI) to the *in situ* released hydrogen peroxide from the HB PEGDA/HA-SH 10.0–1.0 hydrogel containing the lowest amount of GO (25 U/L). As the amount of GO into the hydrogels increased (50–500 U/L), the ZOI values also increased continuously (Figure 7). This means, in detail, that for the sensitive *S*. *aureus* strain, for example, there was a highly significant increase in the ZOI with an increase in GO activity by a factor of five from 25 to 125 U/L (*p* < 0.01). While for all other gram-positive strains, highly significant ZOI enlargements were already shown with a doubling of GO activity from 25 to 50 U/L. A further increase in GO activity from 50 to 125 U/L for the gram-positive strains *S. epidermidis* and MRSA only showed a tendency to increase the ZOI, but this was not significant. A slightly significant increase in ZOI could only be measured for MRSE (*p* < 0.05).

The two tested *P. aeruginosa* strains, the antibiotic-sensitive strain and the highly resistant strain VIM-2-producing drug-resistant *Pseudomonas aeruginosa*, were only susceptible to the hydrogen peroxide amount released from hydrogels with GO activity of 125 U/L. Although a tendency for ZOI to increase was measured for both strains when GO activity was increased from 125 to 250–500 U/L, these were not statistically significant (Figure 8). For the two antibiotic-sensitive strains *E. coli* and *A. baumanii*, a ZOI was only shown from hydrogels with a GO activity of 250 U/L, while for the highly resistant KPC-2-producing drug-resistant *Escherichia coli*, the ZOI was only shown for hydrogels with a GO activity of 500 U/L (Figure 8).

## Discussion

In this work, new antimicrobial hydrogels capable of releasing hydrogen peroxide *in situ* through a honey-inspired approach were designed, to be used as injectable or preformed hydrogels for future applications in the treatment of bacterially colonized or infected wounds. The hydrogels consisted of a chemically cross-linked polymeric network formed by the reaction of HB PEGDA and HA-SH components. HB PEGDA was a synthesized hyperbranched polymer (Figure 1) with a Mw of 16,656 Da, PDI of 1.5, vinyl ratio of 57 mol%, and a branching ratio of 43 mol% (Supplementary Figure S4). Thus the vinyl ratio was in the 40–60 mol% range typically reported for hyperbranched polymers suggesting a high degree of branching (Wang et al., 2015). On the other hand, the relatively high vinyl content (43 mol%) is beneficial for faster polymer cross-linking. The herein used HA-SH was produced and purchased from Blafar Ltd.

The HB PEGDA/HA-SH 10.0–1.0 hydrogel formed through reaction of acrylate groups of HB PEGDA and thiol groups of HA-SH *via* Michael addition reaction. Michael addition works by the weak sulfur-hydrogen bonds of HA-SH reacting with the electron-deficient acrylate groups in the HB PEGDA (Nair et al., 2014) (Figure 2A). The ability to achieve quantitative conversion without by-product formation, even under dilute conditions, makes thiol-Michael addition click reaction particularly suitable for biomedical applications (Nair et al., 2014).

In this work, one HB PEGDA/HA-SH hydrogel composition was explored for the design of injectable hydrogels for *in situ* formation and release of hydrogen peroxide: HB PEGDA/HA-SH 10.0/1.0 hydrogel, based on HB PEGDA/HA-SH blend with 91/9 w/w relative composition, and having 11% w_blend_/v_water_ overall hydrogel concentration. This hydrogel showed a gelation time of around 60 s which ensured injectability. Proof-of-concept hydrogels with different and reproducible shapes were obtained, including hydrogel droplets on Teflon substrates and cylindrical hydrogels through molding (Figures 2B,C). Water uptake tests, performed on small volumes of hydrogels (200 µL) showed their limited swelling ability in the first 2 weeks of incubation in PBS and their resistance to loss of shape up to 64 days (Figure 3). Despite this observation, similar hydrogel systems composed of cross-links of PEG-diacrylates or PEG-diacrylamides with sulfhydryl group-containing peptides are known to exhibit hydrolytic instability not only at pH 8, but also more slowly at pH 7.4. Hydrolysis kinetics show that the hydrolysis half-life is a few days (Elbert and Hubbell, 2001). In this case, it is not the thioether group formed by Michael addition (click reaction) that is hydrolytically unstable, but the ester bond or amide bond located in the neighboring position with the amide bond being more stable than the ester bond. Other authors point out that the stability of such cross-links is strongly dependent on the neighboring groups and on the overall molecular structure of such hydrogel networks (Rydholm et al., 2011; Pérez-Madrigal et al., 2020). In the future, the biodegradability of the HB PEGDA/HA-SH system presented here will need to be investigated for application times greater than 24 h. Nevertheless, all the properties presented here are promising and ensure the possibility of long-term release of encapsulated drugs/molecules in case of drug delivery or long-term support of cells in case of cell delivery therapies. (Wechsler et al., 2019). Progressive increase in water uptake versus time is expected to enlarge hydrogel mesh radius increasing hydrogel permeability, favoring the release of drugs or the exchange of nutrients and metabolites, supporting cell viability in cell therapies, bioprinting applications, and other tissue engineering approaches (Richbourg et al., 2021). Indeed, previously, some of the coauthors employed HB PEGDA/HA-SH hydrogels as injectable systems for adipose stem cell therapy to treat diabetic wounds, preclinically validated in mice (Xu et al., 2018). As a next step, in this work, the hydrogel was proposed as an injectable system for the *in situ* formation and release of the antimicrobial agent hydrogen peroxide. As the antimicrobial mechanism of honey mainly depends on GO enzyme and G, allowing the formation of hydrogen peroxide, varying GO amounts were encapsulated into HB PEGDA component, keeping a fixed G content in the HA-SH component. Hydrogen peroxide production, determined under static conditions for a period of 24 h, increased as a function of GO concentration up to 250 U/L and then settled to an approximately constant value (10.8 ± 1.4 mmol per L hydrogel) (Figure 4). The amount of released H_2_O_2_ can be changed based on hydrogel volume (at fixed GO and G content) or by tuning GO content (at fixed G amount and hydrogel volume). A previous study by Loo et al. showed that daily topical application of 15 µL H_2_O_2_ solution with varying concentrations (10–166 mM) on skin wounds in C57/BL6 mice for overall 10 days caused different effects, from enhanced angiogenesis with 10 mM, i.e., 0.15 µmol/wound, to wound closure retard with 166 mM, i.e., 2.5 µmol/wound, (Loo et al., 2012).

A proper GO amount at constant G content should be selected to provide an antibacterial effect on a skin wound without causing simultaneous damage to dermal fibroblasts of the wound bed and to epidermal keratinocytes from wound edges. Therefore, initially *in vitro* investigations were performed in order to confirm the cytocompatibility of the HB PEGDA/HA-SH 10.0–1.0 hydrogel and to investigate the cytotoxic effect of the released amounts of hydrogen peroxide. Two internationally well-known fibroblast (L929) and epithelial (HaCaT) cell lines were used for the *in vitro* analysis, following DIN EN ISO 10993-5 standards. Furthermore, they represent typical starting cell lines for future investigations in the field of external wound treatment (Rajan et al., 2020; Ribeiro et al., 2020; De la Cruz-Concepción et al., 2021).

Before investigating the antibacterial activity and efficacy of the *in situ* hydrogen peroxide-releasing HB PEGDA/HA-SH hydrogel samples on certain gram-positive and gram-negative bacteria, quantitative direct and indirect cytotoxicity analyses were performed by the CTB assay on L929 and HaCaT cultures. Both tests are part of DIN EN ISO 10993-5 standards. Direct cytotoxicity tests allow the analysis of the behavior of cells in contact with or close to H_2_O_2_-releasing HB PEGDA/HA-SH hydrogel. In this test, results are affected by the release of H_2_O_2_ and the swelling of the hydrogel and its interaction with the cells. However, the direct test cannot separate the cytotoxic effect due to released H_2_O_2_ amount from that due to the hydrogel composition or its acting as a physical barrier in the diffusion of nutrients and catabolites. In contrast, in the indirect cytotoxicity test, the HB PEGDA/HA-SH sample itself is not in contact with the cells, and only H_2_O_2_ released in the medium may affect cell cytotoxicity.

Direct contact of the L929 fibroblasts with the HB PEGDA/HA-SH 10.0–1.0 hydrogels containing different amounts of GO (25–500 U/L) and constant G amount (2.5% w/w) showed a strong decrease in viability with increasing enzyme loading in the quantitative CTB assay (Figure 5A). In contrast, the indirect assay only showed a slight decrease in the viability of the fibroblasts with increasing GO amount (Figure 5B). L929 fibroblast viability decrease in the direct contact assay was probably due to an undersupply of nutrients and oxygen to the cells and additionally to the simultaneous H_2_O_2_ release and its accumulation below the hydrogel sample.

In contrast to fibroblasts, in direct tests, HaCaT keratinocytes showed no decrease in viability with increasing GO loading in the HB PEGDA/HA-SH 10.0–1.0 hydrogel, except for GO equal to 500 U/L, showing nonsignificant decrease in viability (Figures 6A,B). As compared to L929 fibroblasts, HaCaT keratinocytes were resistant to H_2_O_2_-induced cytotoxicity. Such findings are in agreement with *in vitro* data discussed in the study by Lee et al. who also showed that HaCaT keratinocytes gave slightly higher viability values than L929 fibroblasts (Lee et al., 2017).

Cell morphology studies (in direct contact tests) showed analogous differences in sensitivity to oxidative stress by released H_2_O_2_ between L929 fibroblasts and HaCaT keratinocytes when considering the hydrogel samples with 250 and 500 U/L GO activity (Supplementary Figures S5A,B, S6A,B). In the case of L929 cells interaction with hydrogels containing 125 U/L GO, the CTB assay showed a cell viability of 100% compared to those in the TCPS control and almost equal to the unmodified HB PEGDA/HA-SH hydrogel (Figure 5A). The corresponding fluorescence microscopic image after L/D staining suggested a large number of L929 cells showing esterase metabolism and converting calcein-AM into calcein (green fluorescence), and the copresence of cells with compromised membrane integrity allowing the intercalating dye EthD-III to penetrate L929 cells and interact with their DNA (red fluorescence) (Supplementary Figure S5B, 125 U/L). This finding suggested that the evaluation of real cytotoxic data is not sufficient by performing only one assay or measurement principle.

Figure 7 demonstrates the antibacterial activity of *in situ* H_2_O_2_-releasing HB PEGDA/HA-SH 10–1.0 hydrogels containing different GO activities (25–500 U/L) at constant G amount of 2.5 wt% against the gram-positive bacteria *S. aureus* and *S. epidermis* and two antibiotic resistant clinical isolates of MRSA and MRSE. In agar diffusion assays, ZOI—measured on hydrogel samples with the same volume (30 µL) as the ones used for cytocompatibility assays—increased with increasing the GO concentration and was also displayed in the case of methicillin-resistant gram-positive bacteria. However, in contrast to gram-positive bacteria, gram-negative bacteria *P. aeruginosa* and VIM-2-producing drug-resistant *P. aeruginosa* only showed ZOI at GO activity ≥125 U/L (Figure 8). For an antibiotic-sensitive *E. coli* and *A. baumanii* strain, the ZOI was only measured in the case of hydrogels with GO activity of 250 U/L. In addition, ZOIs of the hydrogel samples with higher GO activities were large for gram-positive compared to gram-negative bacteria, indicating that the gram-positive bacteria are much more susceptible to oxidative stress. There are various reasons for this significant difference between the measured ZOI for gram-positive compared to the gram-negative bacteria. One reason is the different structural composition of gram-positive and gram-negative bacteria: gram-negative bacteria have an outer lipid-based membrane that is additionally decorated with lipopolysaccharides (LPS), while gram-positive bacteria lack this outer lipid bilayer. The peptidoglycans of the outer layer of the gram-positive bacteria have been reported to be significantly more sensitive to oxidation (Rapacka-Zdończyk et al., 2021). In addition, Lee et al. showed that the LPS of the gram-negative bacteria have an antioxidant effect and thus eliminate part of the hydrogen peroxide produced and released *in situ* (Lee et al., 2017).

## Conclusion

In summary, an *in situ* producing H_2_O_2_-PEGDA/HA-SH 10–1.0 hydrogel was developed that can be used as a drug-free antibacterial injectable hydrogel for the treatment of infected wounds. Based on the data so far, it can be said that GO activities of 125 U/L resulted in acceptable ZOIs for the gram-positive bacterial strains without affecting the viability of the fibroblasts too much within 24 h. This is a good indication that the hydrogel can be used for the treatment of infected wounds.

It was also shown that the keratinocytes were significantly less sensitive. To the extent possible based on this initial *in vitro* study, this honey-mimetic antibacterial hydrogel could also be used as a complementary system with some gram-negative bacteria such as *P. aeruginosa* and VIM-2-producing drug-resistant *P. aeruginosa*. Here, further studies with bacterially colonized 3D skin equivalents and over longer cultivation periods are required to assess the effective extent of cytotoxicity of the hydrogen peroxide released from this hydrogel. It is expected that the use of alternative antimicrobial solutions to antibiotics will help combat antimicrobial resistance. Considering its rapid formation, ability to produce H_2_O_2_ continuously, and antibacterial activity against susceptible and methicillin-resistant gram-positive bacteria while being cytocompatible for eukaryotic cells, this type of hydrogel represents a promising material for wound dressings and deserves further investigation.

## Data Availability

The raw data supporting the conclusion of this article will be made available by the authors, without undue reservation.

## Supplementary Material

Supplementary Figure 1 Preparation of H_2_O_2_-releasing 10% HB-PEGDA/1% HA-SH hydrogel solution.

Supplementary Figure 2 Colorimetric measurement of *in situ* generated H_2_O_2_.

Supplementary Figure 3 Calibration curve of H_2_O_2_ obtained by the pertitanic acid assay.

Supplementary Figure 4
^1^H NMR spectrum of synthesized HB PEGDA (Mw 16,656 Da).

Supplementary Figure 5
**(A)** Bright-field micrographs of L929 cells after 24 h interaction with H_2_O_2_-releasing HB PEGDA/ HA-SH 10.0–1.0 hydrogels containing different amounts of GO (25–500 U/L) and constant G amount (2.5% w/w) versus controls (TCPS and lysis controls). **(B)** Fluorescent micrographs of Live/Dead-stained L929 cells after 24 h interaction with H_2_O_2_-releasing HB PEGDA/ HA-SH 10.0–1.0 hydrogels containing different amounts of GO (25–500 U/L) and constant G amount (2.5% w/w) versus controls (TCPS and lysis controls).

Supplementary Figure 6
**(A)** Bright-field micrographs of HaCaT cells after 24 h interaction with H_2_O_2_-releasing HB PEGDA/ HA-SH 10.0–1.0 hydrogels containing different amounts of GO (25–500 U/L) and constant G amount (2.5% w/w) versus controls (TCPS and lysis controls). **(B)** Fluorescent micrographs of Live/Dead-stained HaCaT cells after 24 h interaction with H_2_O_2_-releasing HB PEGDA/ HA-SH 10.0–1.0 hydrogels containing different amounts of GO (25–500 U/L) and constant G amount (2.5% w/w) versus controls (TCPS and lysis controls).
