# Supplementary material for: In situ Forming Hyperbranched PEG—Thiolated Hyaluronic Acid Hydrogels With Honey-Mimetic Antibacterial Properties
Source: Front Bioeng Biotechnol. 2021 Nov 16;9:742135. doi: 10.3389/fbioe.2021.742135 (PMC8637896; doi:10.3389/fbioe.2021.742135)
Supplement: Supplementary file 1 [file DataSheet1.pdf]

Supplementary figures:

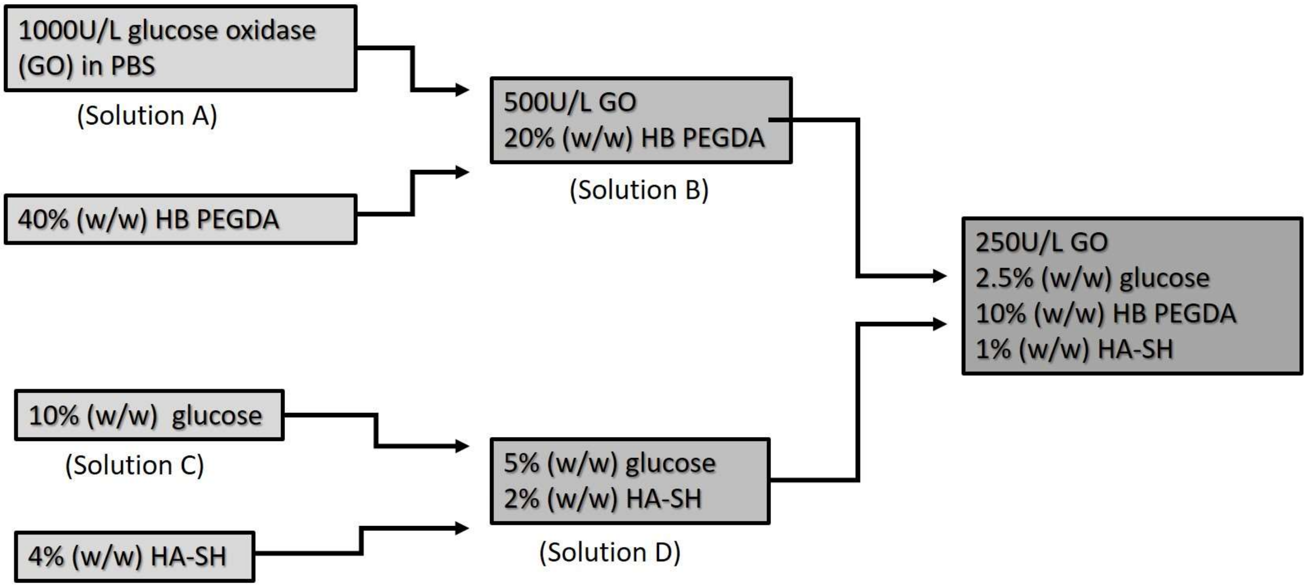

Figure S1. Preparation of H_2O_2 releasing 10% HB-PEGDA/1% HA-SH hydrogel solution.

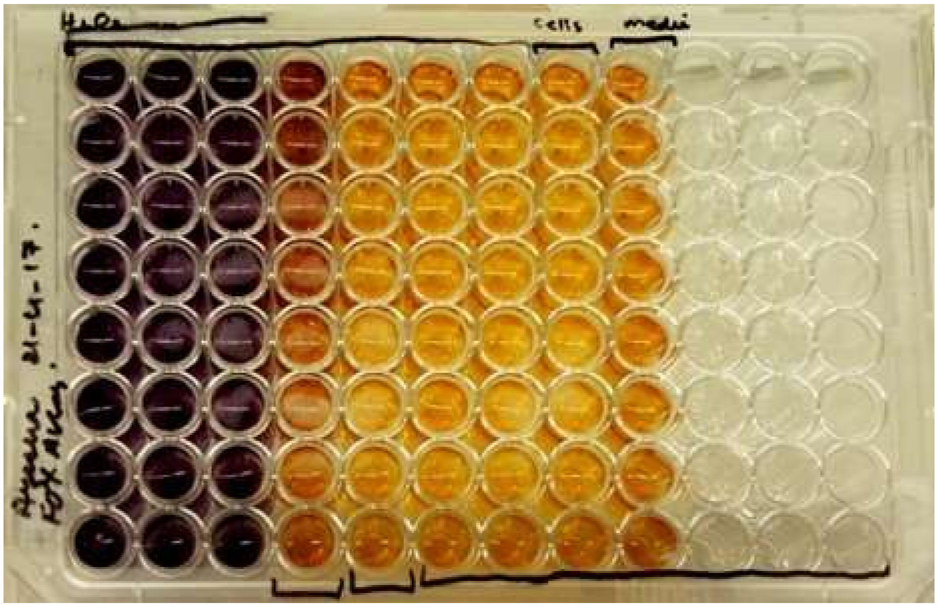

Figure S2. Colorimetric measurement of *in situ* generated H_2O_2.

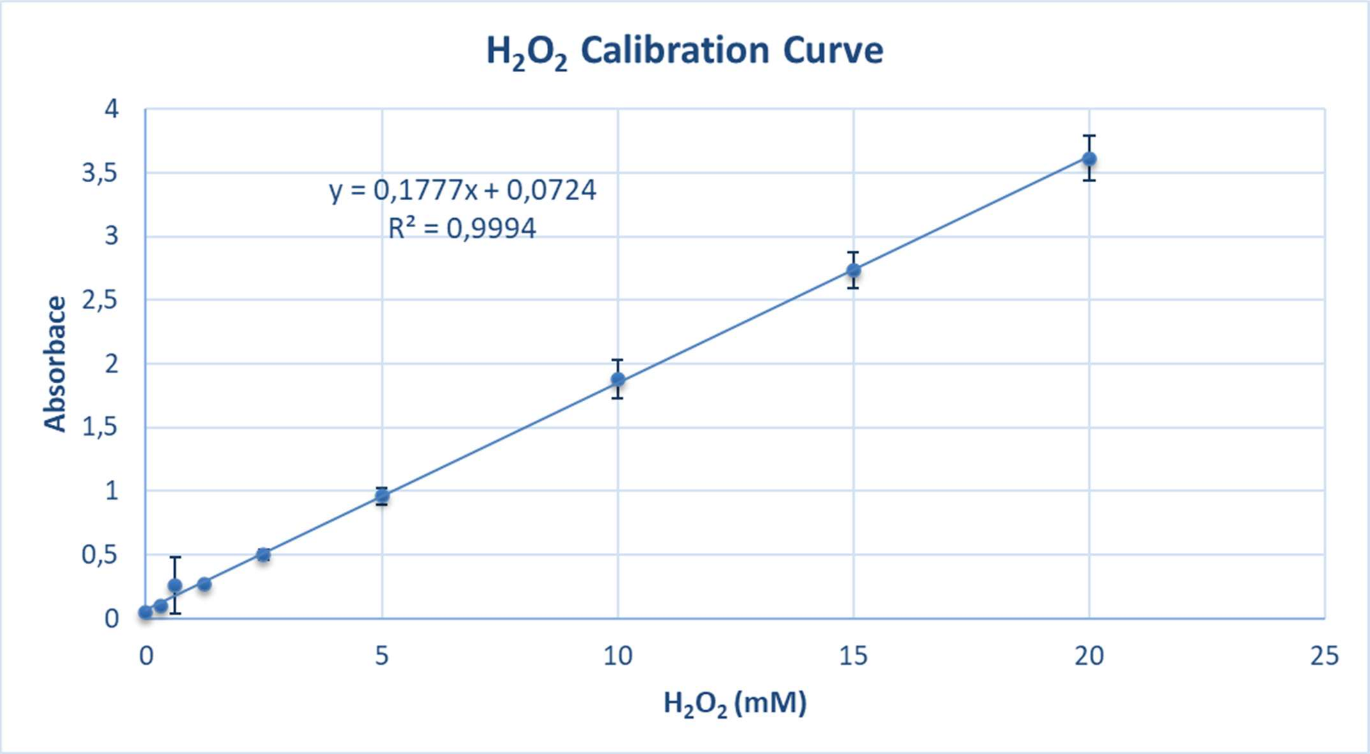

Figure S3. Calibration curve of H_2O_2 by the pertitanic acid assay

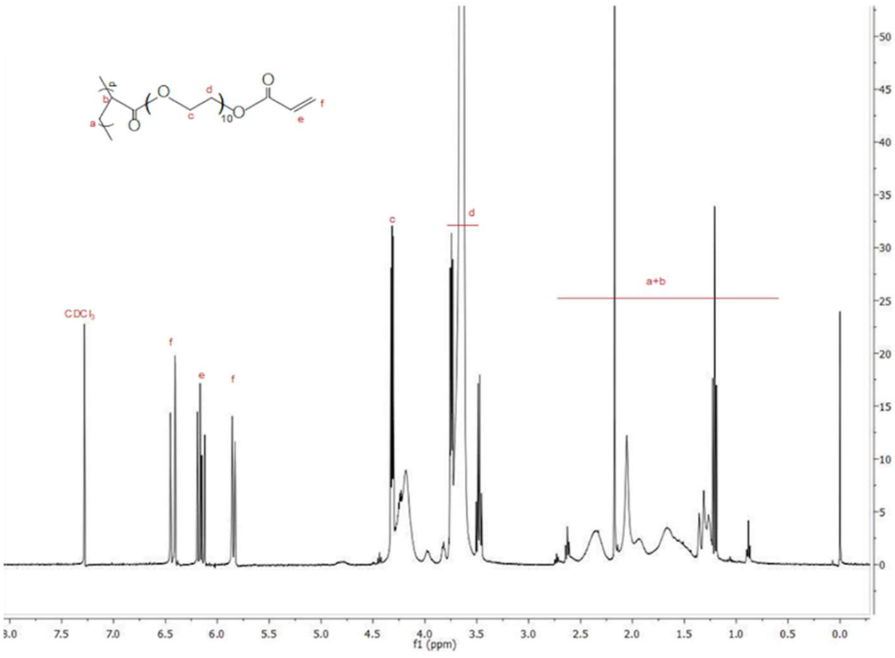

Figure S4. ^{1}H NMR spectrum of synthesized HB PEGDA (Mw 16,656 Da).

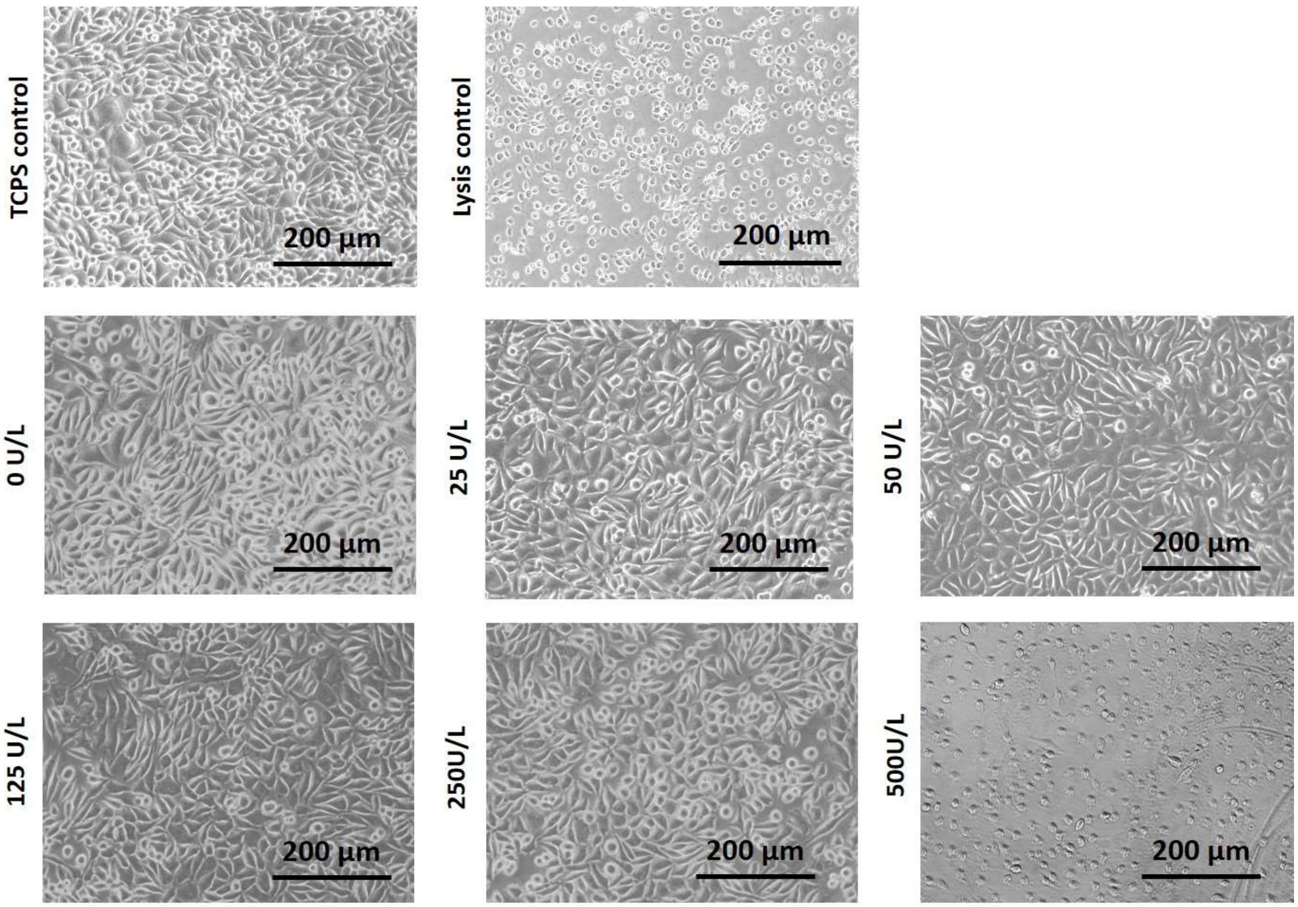

Figure S5A. Bright-field micrographs of L929 cells after 24 h interaction with H_2O_2 releasing HB PEGDA/ HA-SH 10.0-1.0 hydrogels containing different amounts of GO (25 U/L – 500 U/L) and constant G amount (2.5% w/w) versus controls (TCPS and lysis controls).

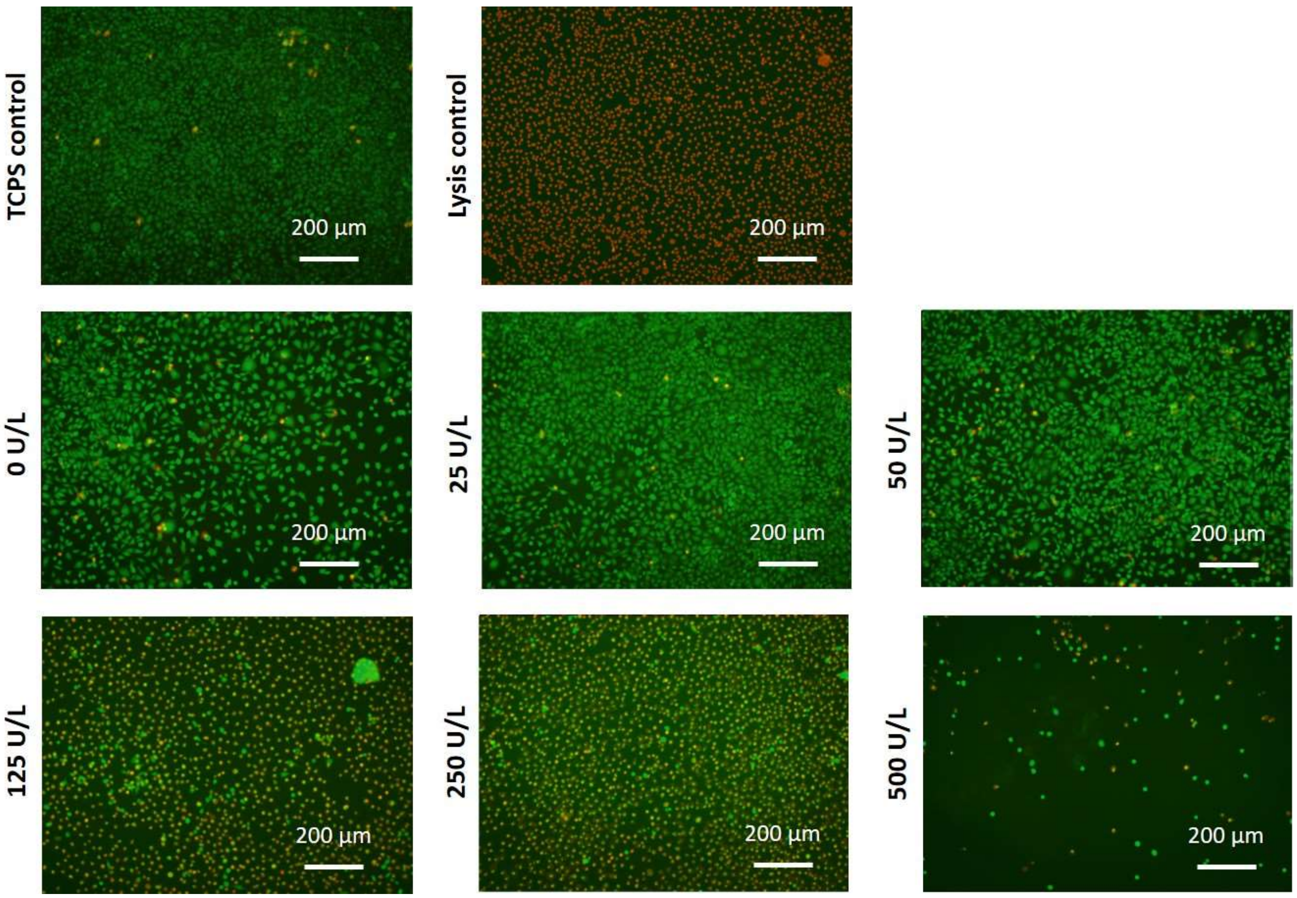

Figure S5B. Fluorescent micrographs of Live/Dead-stained L929 cells after 24 h interaction with H_2O_2 releasing HB PEGDA/ HA-SH 10.0-1.0 hydrogels containing different amounts of GO (25 U/L – 500 U/L) and constant G amount (2.5% w/w) versus controls (TCPS and lysis controls).

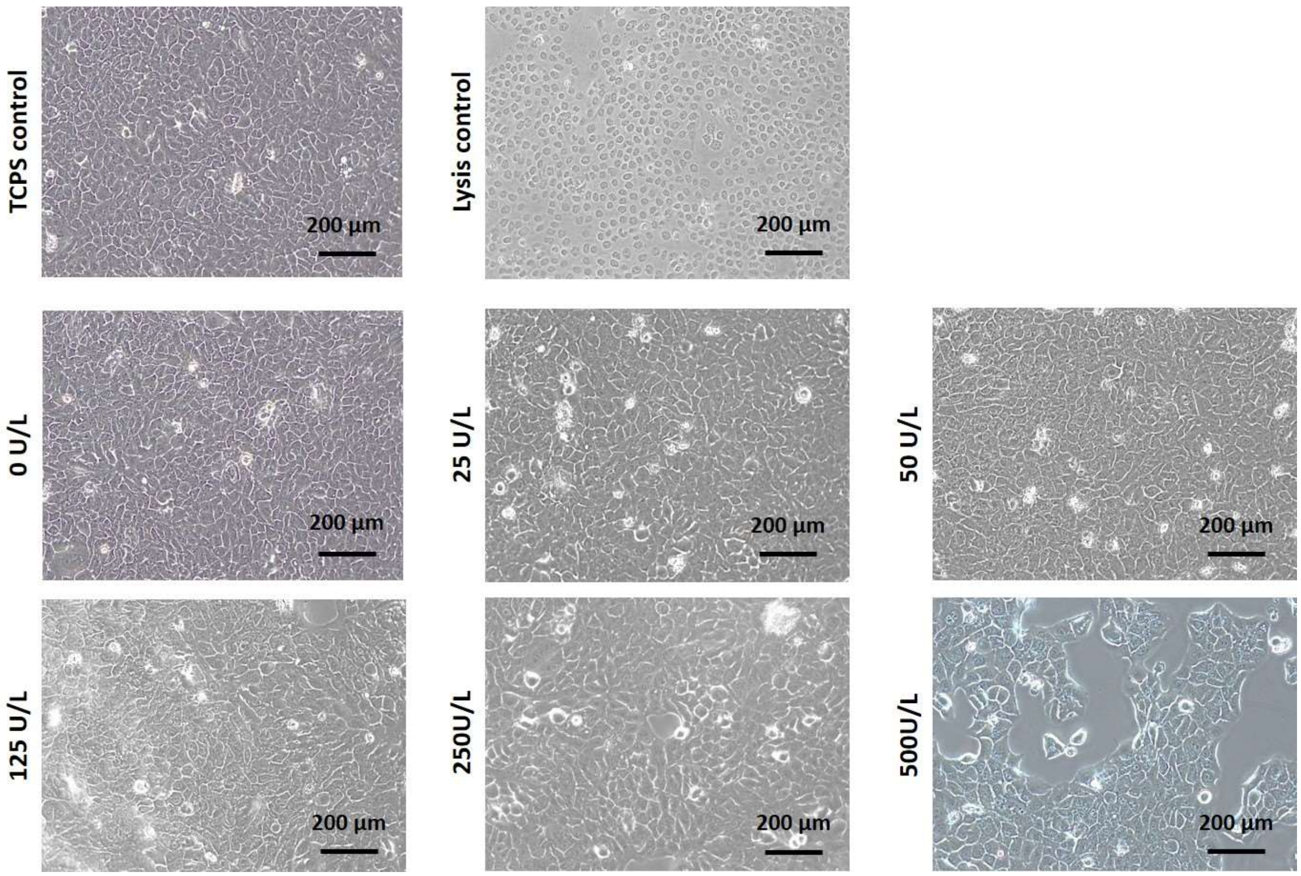

Figure S6A. Bright-field micrographs of HaCaT cells after 24 h interaction with H_2O_2 releasing HB PEGDA/ HA-SH 10.0-1.0 hydrogels containing different amounts of GO (25 U/L – 500 U/L) and constant G amount (2.5% w/w) versus controls (TCPS and lysis controls).

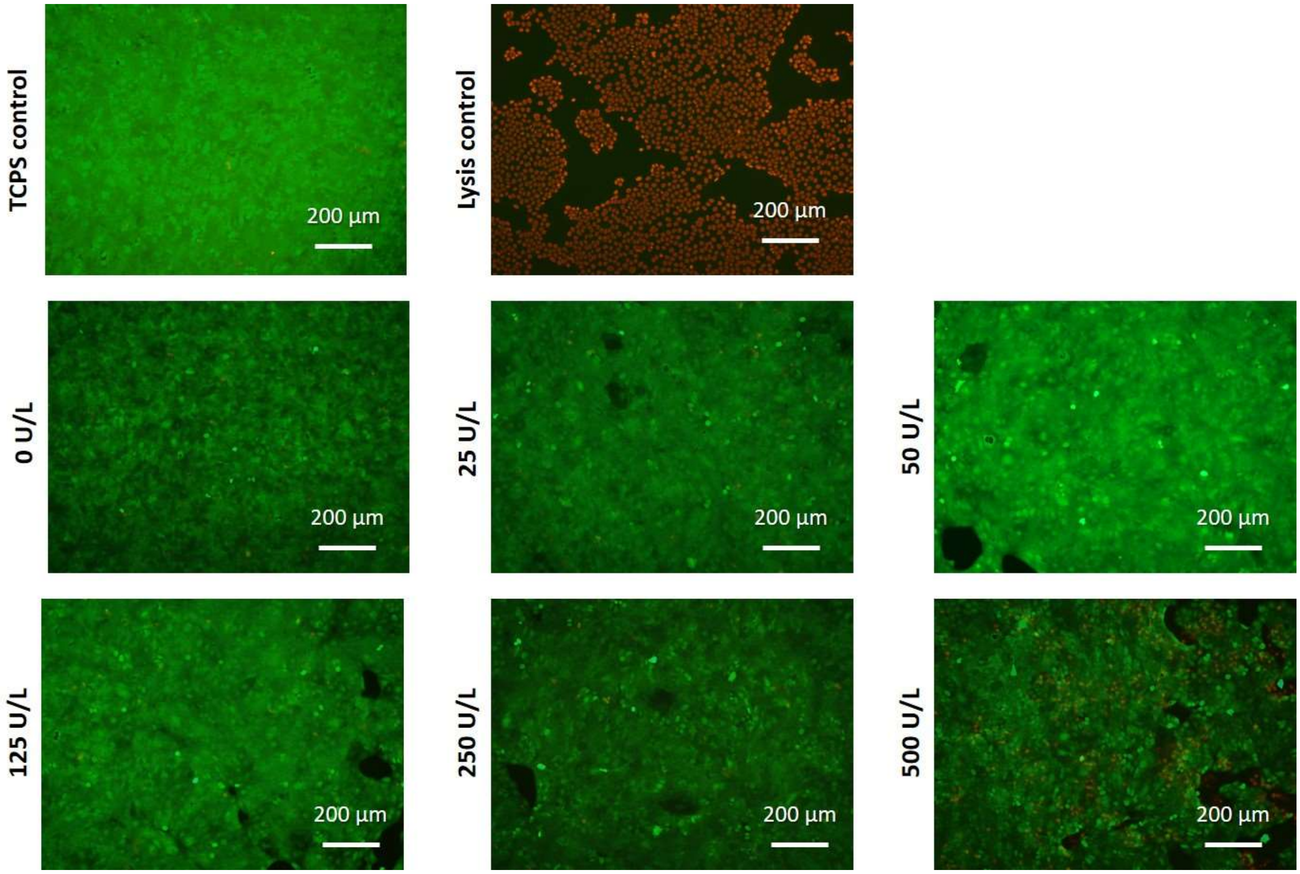

Figure S6B. Fluorescent micrographs of Live/Dead-stained HaCaT cells after 24 h interaction with H_2O_2 releasing HB PEGDA/ HA-SH 10.0-1.0 hydrogels containing different amounts of GO (25 U/L – 500 U/L) and constant G amount (2.5% w/w) versus controls (TCPS and lysis controls).
